# Triple-Probiotic-Fermented Goji (*Lycium barbarum* L.) Ameliorates Metabolic Disorders Associated with Hyperuricemia in Mice

**DOI:** 10.3390/microorganisms13061367

**Published:** 2025-06-12

**Authors:** Lu Ren, Yuechan Li, Shiting Liu, Xiaoke Jia, Hongpeng He, Feiliang Zhong, Fuping Lu, Xuegang Luo

**Affiliations:** Key Laboratory of Industrial Fermentation Microbiology of the Ministry of Education and Tianjin Key Laboratory of Industrial Microbiology, College of Biotechnology, Tianjin University of Science and Technology, Tianjin 300457, China; renlu@mail.tust.edu.cn (L.R.);

**Keywords:** goji (*Lycium barbarum* L., wolfberry), hyperuricemia, fermented beverages, combined probiotics, flavor compounds, intestinal flora

## Abstract

Hyperuricemia (HUA) is a metabolic disorder characterized by excessive uric acid (UA) production and impaired excretion. Goji, as a representative medicinal food, holds significant research and development value, while probiotic fermentation technology is finding increasingly widespread applications in the functional food sector. This study developed a novel goji fermented with three probiotic strains (*Lactoplantibacillus plantarum* CGMCC8198, *Lactococcus lactis* LTJ28, and *Lactocaseibacillus casei* YR2-2) and investigated its anti-HUA effects. Optimal fermentation conditions (7.913 material–liquid ratio, 3.92% inoculation, 7.49 h at 37 °C with 1:1:2 strain ratio) yielded a beverage with enhanced flavor profiles (19 aroma compounds) and high viable counts. In HUA cell models, the 15% fermented goji juice significantly reduced UA levels by 56% (*p* < 0.01). In potassium oxonate-induced HUA mice, the beverage effectively lowered serum UA, xanthine oxidase activity, and renal function markers (blood urea nitrogen and creatinine, *p* < 0.0001) while improving hepatic parameters (alanine aminotransferase, aspartate Aminotransferase). The goji-fermented juice significantly reduced the expression of renal UA transporters GLUT9 and URAT1 (*p* < 0.0001) while improving gut microbiota composition, as evidenced by increased beneficial SCFAs (acetic acid, butyric acid, *p* < 0.0001) and elevated Lactobacillus abundance 2.14-fold. Our findings demonstrate that this triple-probiotic-fermented goji beverage represents an effective dietary strategy for HUA management by simultaneously inhibiting UA production, enhancing excretion, and restoring gut microbiota homeostasis, providing a scientific basis for developing probiotic-based functional foods against HUA.

## 1. Introduction

Hyperuricemia (HUA) is a metabolic disease caused by a disorder of purine metabolism and an imbalance in the formation and secretion of uric acid (UA). Chronic HUA may cause metabolic syndrome and gout, damage the kidneys, and is also associated with many health problems, such as cardiovascular disease, as well as affecting the balance of intestinal microorganisms in vivo and the integrity of intestinal epithelium [1]. Current therapeutic strategies for HUA primarily focus on pharmacological interventions, such as xanthine oxidase (XOD) inhibitors (e.g., allopurinol) and uricosuric agents (e.g., probenecid). However, these treatments are often associated with adverse effects, including hypersensitivity reactions, gastrointestinal disturbances, and renal toxicity [2]. Consequently, there is a growing interest in exploring alternative or adjunctive therapies, particularly those derived from natural sources, to manage HUA effectively and safely.

In recent years, probiotics have emerged as promising candidates for modulating metabolic disorders, including HUA. Probiotics are considered potential functional foods; they not only enhance the nutritional value and flavor of foods but also help to maintain the stability of intestinal flora [3]. Fermented foods with added probiotics occupy an important position in modern diets, such as the application of probiotics such as *Lactobacillus bulgaricus* and *Bifidobacterium bifidum* in the production of dairy products and the application of *lactobacilli* in fermented fruit and vegetable juices [4]. Among the various probiotic strains, *Lactoplantibacillus plantarum* (previously *Lactobacillus plantarum*), *Lactococcus lactis,* and *Lactocaseibacillus casei* (previously *Lactobacillus casei*) have garnered significant attention due to their well-documented health-promoting properties, including anti-inflammatory, antioxidant, and immunomodulatory effects [5]. These strains have been extensively studied for their ability to modulate gut microbiota, enhance intestinal barrier function, and regulate host metabolism [6,7]. Notably, emerging evidence suggests that gut microflora and certain probiotic strains can influence purine metabolism and uric acid excretion, potentially offering a novel approach to managing HUA [8]. *L. plantarum* has been shown to exert beneficial effects on lipid metabolism, glucose homeostasis, and oxidative stress [9]. Its ability to produce bioactive peptides and short-chain fatty acids (SCFAs) may contribute to its metabolic regulatory effects [10]. *L. lactis* has demonstrated the potential to reduce systemic inflammation and improve gut health. Its capacity to produce beneficial compounds, such as exopolysaccharides (which can modulate immune responses and protect the gut barrier) and bacteriocins (antimicrobial peptides that inhibit pathogenic bacteria), could further enhance its therapeutic potential [11,12]. *L. case* has been reported to alleviate components of metabolic syndrome, such as obesity and insulin resistance, and possesses immunomodulatory effects [13]. These three strains, when used in combination, may offer a multifaceted approach to addressing the complex pathophysiology of HUA.

As a traditional medicinal and edible plant with a history spanning thousands of years, goji (*Lycium barbarum* L., wolfberry) is recognized for its rich nutritional composition and potential health benefits [14]. Modern pharmacological research has shown that goji berries are rich in lycium polysaccharides, betaine, ascorbic acid, and other trace elements, which can improve cardiovascular disease, reproductive organ damage, Alzheimer’s disease, and other diseases [15]. Recent studies have suggested that goji consumption may improve lipid profiles, enhance antioxidant capacity, and modulate gut microbiota composition [16]. In previous studies in our laboratory, probiotics with uric acid-lowering function have been screened from naturally fermented goji, and the mechanism of uric acid-lowering effect of this strain of *Pediococcus acidilactici* GQ01 and its postbiotic elements on HUA mice and the composition of intestinal microorganisms have been investigated [17]. The combination of fermented foods and probiotics not only enhances the nutritional value of the food but also enhances its positive impact on health, including improving gut health, boosting immunity, and providing essential nutrients [10]. Given its potential to synergize with probiotics, goji represents an ideal substrate for developing functional fermented beverages aimed at managing metabolic disorders, including HUA.

Multi-probiotic-fermented functional beverages demonstrate specific metabolic disease-modulating effects through their synergistic microbial activities and bioactive compound profiles [11]. They help to provide probiotics and maintain a healthy microbiota with significant effects on the immune system and metabolic function [18,19]. Despite the promising potential of probiotic-fermented goji beverages in managing HUA, there is a paucity of research investigating the specific effects of *L. plantarum*, *L. lactis,* and *L. casei* co-fermented goji on UA metabolism. Existing studies have primarily focused on the individual effects of these strains or their application in other metabolic contexts. The rationale for using a multi-strain probiotic approach lies in the potential for combined effects of the strains, leading to enhanced metabolic effects [20]. In this study, we established the fermentation process of goji beverage using three probiotic strains (*L. plantarum* TCCC11824, *L. lactis* LTJ28, and *L. casei* YR2-2), and investigated the therapeutic effects of both conventional goji juice and the probiotic-fermented beverage on HUA using cellular and murine models, with particular emphasis on UA metabolism and intestinal flora modulation.

## 2. Materials and Methods

### 2.1. Reagents and Materials

The dried goji berries were collected and purchased in Zhongning County, Ningxia Hui Autonomous Region, China. *L. plantarum* TCCC11824 (CGMCC NO. 8198) [21], *L. lactis* LTJ28 (CGMCC NO. 20087) [22], *L. casei* YR2-2, and HepG2 cells, all of which were preserved in Tianjin University of Science and Technology.

### 2.2. Strain Culture and Preparation of Goji-Fermented Beverage

The strains were inoculated into MRS liquid medium at a 1% inoculum for activation, cultured in a biochemical incubator at 37 °C, and passaged three times during the exponential period to activate to a viable count of more than 10^8^ CFU/mL. The goji beverage was pasteurized, and a triple-probiotic combination consisting of *L. plantarum* TCCC11824, *L. lactis* LTJ28, and *L. casei* YR2-2 was inoculated into the sterilized and cooled goji beverage for fermentation.

### 2.3. Detection of Antioxidant Activity of Probiotic-Fermented Goji Beverage

A 0.2 mmol/L DPPH working solution was prepared using anhydrous ethanol as the solvent. A 1.0 mL aliquot of the fermentation sample was mixed with 1.0 mL of the DPPH solution and 3.0 mL of deionized water in a test tube. After thorough vortexing, the mixture was incubated in darkness for 30 min at room temperature. The absorbance was measured at 517 nm using a spectrophotometer. The radical scavenging activity was calculated using the following formula:

Clearance Rate = [1 − (A_X_ − A_X0_)]/A_0_ × 100% (1)

A_X_: 1.0 mL fermentation solution + 1.0 mL DPPH+ 3.0 mL distilled water;

A_0_: 1.0 mL DPPH+ 4.0 mL distilled water;

A_X0_: 5.0 mL anhydrous ethanol.

### 2.4. Single-Factor Experiment on Goji Fermentation

We determined the following parameters and ratios based on the results of the preliminary experiment. The fermentation process of goji juice was optimized through single-factor experiments, focusing on five key parameters: liquid-to-solid ratio (2, 4, 6, 8, 10), fermentation time (4, 6, 8, 10, 12 h), fermentation temperature (31, 34, 37, 40, 43 °C), probiotic strain ratio (*L. plantarum* TCCC11824: *L. lactis* LTJ28: *L. casei* YR2-2 at 1:1:1, 2:1:1, 1:2:1, 1:1:2, 3:1:1), and inoculation concentration (1, 2, 3, 4, 5%). Based on antioxidant activity and viable cell count (Table 1), the three most influential factors were identified for subsequent response surface methodology (RSM) optimization to determine the optimal fermentation conditions.

### 2.5. Optimization of Goji Fermentation Process Using Response Surface Analysis

To quantify viable bacteria in probiotic-fermented goji beverages, serial 10-fold dilutions were prepared. Three serial ten-fold dilutions (10^−8^, 10^−9^, 10^−10^ CFU/mL) were prepared, and 100 μL aliquots were plated in triplicate on MRS agar. After incubation at 37 °C for 36 h, colony-forming units were enumerated. The fermented beverages were evaluated according to the scoring criteria in Table 2. Significant factors were identified through single-factor experiments, and their optimal ranges were determined based on the comprehensive scores of antioxidant activity and viable cell counts (Table 1). Subsequently, the RSM experimental design was established using statistical software, with the detailed design presented in Table 2.

### 2.6. Determination of Flavor Substances in Fermented Beverages by SPME-GC/MS

For the analysis of volatile compounds in goji juice and goji-fermented beverage, a solid-phase microextraction (SPME) combined with gas chromatography–mass spectrometry (GC–MS) method was employed [23]. Five milliliters of each sample were transferred into sample vials and equilibrated in a 40 °C water bath for 15 min to homogenize the volatile distribution. An SPME fiber was then inserted into the vial to adsorb volatile substances for 30 min. In the GC–MS analysis, the GC oven temperature was initially set at 40 °C for 2 min, then increased to 150 °C at a rate of 4 °C/min, and held for 2 min, followed by a final increase to 250 °C at 8 °C/min and held for 6 min. The MS quadrupole temperature was set at 250 °C, and the ion source temperature was set at 280 °C. After the analysis, the obtained mass spectra were compared with the NIST general database. Compounds with a matching degree > 98% were selected to identify the types and determine the relative contents of alcohols, aldehydes, ketones, acids, and other compounds in the samples.

### 2.7. Cell Viability Assay and Construction of Cellular Models

HepG2 cells meeting viability and quality criteria (>95% viability, displaying typical morphology, and showing no contamination signs) were seeded into 24-well plates and cultured in a cell incubator until adherent. The cells were treated with basic culture medium or goji-fermented beverages at different concentrations for 24 h. Subsequently, 2 μL of 2.5 mol/L adenosine was added to the medium and incubated for another 24 h. Finally, 0.46 μL of xanthine oxidase was added, followed by an 8 h incubation. UA levels were then measured according to the UA detection kit instructions.

### 2.8. Animals and Treatment

The animal experiments were approved by the Ethics Committee of Tianjin University of Science and Technology and conducted in accordance with the guidelines in the Manual for the Treatment and Utilization of Laboratory Creatures (ethics number: SWKL20231116). Fifty 4-week-old male Kunming mice were obtained from SPF Biotechnology Co., Ltd. (Beijing, China) (Quality Certificate SCXK (JING) 2019-0010). All mice were kept in a constant environment (24 ± 2 °C, 52 ± 5% humidity, 12 h light–dark cycle) with free access to water and a normal diet. The mice were randomly divided into 5 groups (*n* = 10) after 1 week of co-housing, as follows: normal control group (NC), HUA group (M), goji juice group (GJ), triple-probiotic-fermented goji beverage group (TR, with *L. plantarum* TCCC11824, *L. lactis* LTJ28, and *L. casei* YR2-2), and positive-control allopurinol group (AP). The HUA mouse model was established by intraperitoneal injection of 300 mg/kg·d potassium oxazinate and a high-purine diet (10 g/kg·d yeast paste). Mice in the NC group received 0.2 mL/d of normal saline, while those in the M group received the same amount of normal saline along with the HUA-induction treatment. Mice in the GJ group were given 0.2 mL/d goji juice; the TR group received 0.2 mL/d triple-probiotic-fermented goji beverage; and the AP group was given 30 mg/kg·d allopurinol for 28 days. All administrations were performed via oral gavage. After fasting for 12 h, the mice were euthanized 1 h after the last administration of treatments. Blood was obtained from the eye sockets, and serum was obtained via centrifugation for 10 min at 4 °C and 3000 rpm. The liver and kidney tissues were dissected and weighed, and the liver, kidney, small intestine, and colon contents were rapidly frozen in liquid nitrogen and stored at −80 °C for further analysis. The samples were tested for UA, BUN (Urea nitrogen), CRE (Creatinine), ALT (Albuminous transaminase), AST (Albuminous transaminase), and XOD (Xanthine oxidase) activity using a Nanjing JianCheng Bioengineering Institute kit (Nanjing, China).

### 2.9. Histological Analysis of Kidney and Small Intestine Tissues

Kidney and small intestine tissues were dehydrated and preserved in 4% paraformaldehyde, embedded in paraffin, and then stained with hematoxylin and eosin (H&E). The slides were observed in a blinded fashion with the utilization of an orthogonal fluorescence microscope (Olympus BX53, Tokyo, Japan). At least 3 samples per group from each section were randomly selected for analysis at 200× and 100× magnification. Renal tubules with the following histopathological changes were determined as injured: tubular dilatation, irregular lumen shape, flattening, and sloughing of renal tubular epithelial cells. Pathological damage was scored 0–4 grades by the percentage of injured tubules (0: no injury; 1: mild < 25%; 2: moderate < 50%; 3: severe < 75%; 4: very severe > 75%). The scoring corresponds to intestinal morphology as follows: 0, normal villi; 1, twisted villus tips; 2, goblet cell and crypt disappearance; 3, patchy epithelial cell sloughing; 4, exposed villi, intact lamina propria, and epithelial cell shedding; 5, exposed lamina propria; and 6, villous hemorrhage or sloughing. Sixty villi per group were scored, and the average score was used for statistical analysis.

### 2.10. Quantitative Real-Time Polymerase Chain Reaction (qRT-PCR) Analysis

Total RNAs were extracted from mouse kidney tissues using Trizol reagent (Takara, Kyoto, Japan). Subsequently, 2 mg of the extracted RNA was reverse-transcribed into cDNA with the LunaScript RT SuperMix Kit (NEB, Ipswich, MA, USA) following the manufacturer’s instructions. For gene expression analysis, RT-PCR was conducted utilizing the SYBR Green Premix Pro Taq HS qPCR kit from Accurate Biotechnology (Guangzhou, China). All gene expression levels were then normalized to those of β-actin. All primers (Table 3) used in this study were synthesized by GENEWIZ Biotechnology Co. (Tianjin, China). The relative mRNA levels of the PCR products were quantified using β-actin as an internal standard and calculated with the 2^−ΔΔCt^ method.

### 2.11. Gut Microbiota Analysis

The mouse colon contents were sent to Majorbio Technology Co. (Shanghai, China) for 16S rDNA sequencing. The following procedures were sequentially performed: DNA extraction from the mouse colon contents, PCR amplification, construction of the sequencing library by ligating sequencing adapters, and high-throughput sequencing. The V3-V4 region of 16S rDNA was amplified using specific primers 338F (5′-ACTCCTACGGGGAGGCAGCAG-3′) and 806R (5′-GGACTACHVGGGTWTCTAAT-3′). The UPARSE pipeline was employed to cluster valid tags into operational taxonomic units (OTUs) with a minimum similarity of 97%. The sequencing data were further analyzed for interactions. Based on the OTUs in the mouse intestine, α-diversity analysis, β-diversity analysis, hierarchical clustering analysis, principal coordinate analysis, and species difference analysis were carried out.

### 2.12. Analysis of SCFAs via Gas Chromatography (GC)

GC (Agilent GC7890A, Santa Clara, CA, USA) was employed to detect SCFAs in the mouse colon contents. The parameters of the instrument were set as follows: an injection volume of 1 µL, an inlet temperature of 250 °C, a split ratio of 1:1, a chromatographic column of HP-5 (19091J-413) with dimensions of 30 cm length, a 320 µm inner diameter, and a column flow rate of 2 mL/min. The initial column temperature was set at 90 °C, held for 6 min, and then increased to 200 °C at a rate of 10 °C per minute. The detector heater was set to 250 °C, with an H_2_ flow rate of 30 mL/min and an airflow rate of 300 mL/min.

### 2.13. Statistical Analysis

Data were analyzed using SPSS statistical software 23.0 with one-way ANOVA and Duncan post-hoc test, Design-Expert response surface analysis software for processing and statistical analysis. Graphing was performed using GraphPad Prism 8.0 software. Quantitative data were expressed as mean ± standard deviation (SD). *p* < 0.05 was considered statistically significant.

## 3. Results and Discussion

### 3.1. Optimization of Fermented Goji Beverage by Triple Probiotics

This experiment aimed to separately examine the impacts of material–liquid ratio, fermentation time, fermentation temperature, inoculation amount, and the proportion of the triple-probiotic on both the antioxidant capacity and the viable probiotics count of the fermented goji beverage. Beverages with high antioxidant activity can scavenge free radicals and reduce oxidative stress, thus helping to prevent many chronic diseases [24]. A high viable count means that the beverage has a stronger probiotic effect, which can provide more health benefits to consumers, such as improved intestinal health and enhanced immunity [17].

**Figure 1:**
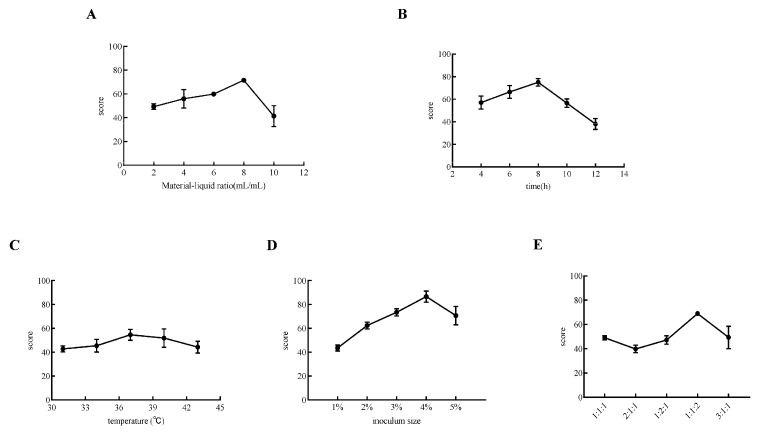
Influence of single-factor triple-probiotic fermentation on the comprehensive score of goji-probiotic-fermented beverages. (**A**) Material–liquid ratio; (**B**) Fermentation time; (**C**) Fermentation temperature; (**D**) Strain inoculation amount; (**E**) Ratio of triple-probiotic.

As shown in Figure 1, the influence of all the investigated factors on the score of fermented beverages presented a parabolic pattern. Specifically, when the liquid–liquid ratio was set at 8 mL/mL, the comprehensive score, which was composed of antioxidant capacity and the viable bacterial count, reached its peak (Figure 1A). The highest score was obtained when the fermentation time was 8 h (Figure 1B). This phenomenon might be attributed to the fact that if the fermentation time is too short, the probiotics in the beverage have not yet entered the logarithmic growth phase. On the contrary, an overly long fermentation time could cause the probiotics in the beverage to enter the plateau growth phase. Regarding the fermentation temperature, it appeared that 37 °C was the most optimal choice (Figure 1C). In terms of the fermentation strains, the most appropriate inoculation amount for the triple-strain mixture was 4%, and the optimal ratio for *L. plantarum* CGMCC8198, *L. lactis* LTJ28, and *L. casei* YR2-2 should be 1:1:2.

Subsequently, in order to further explore the comprehensive impact of the three key factors (fermentation time, strain inoculation amount, material–liquid ratio) obtained in the single-factor experiment on the beverage fermentation, the response surface optimization analysis was performed. The design scheme and results of the response surface analysis are shown in Table 4.

Moreover, through further analysis by the Design-Expert.10 software, it was found that the multivariate quadratic regression equation of the fermented beverage scores with the three factors was Y = 82.04 − 3.10A − 1.07B − 0.075C + 0.5AB + 1.5AC − 1.45BC − 6.4A^2^ − 7.15B^2^ − 3.84C^2^. In addition, the significance of the tested results of model regression coefficients is shown in Table 5; the impact order of the three factors was A > B > C, indicating that fermentation time had a more significant effect on beverage fermentation.

**Figure A1:**
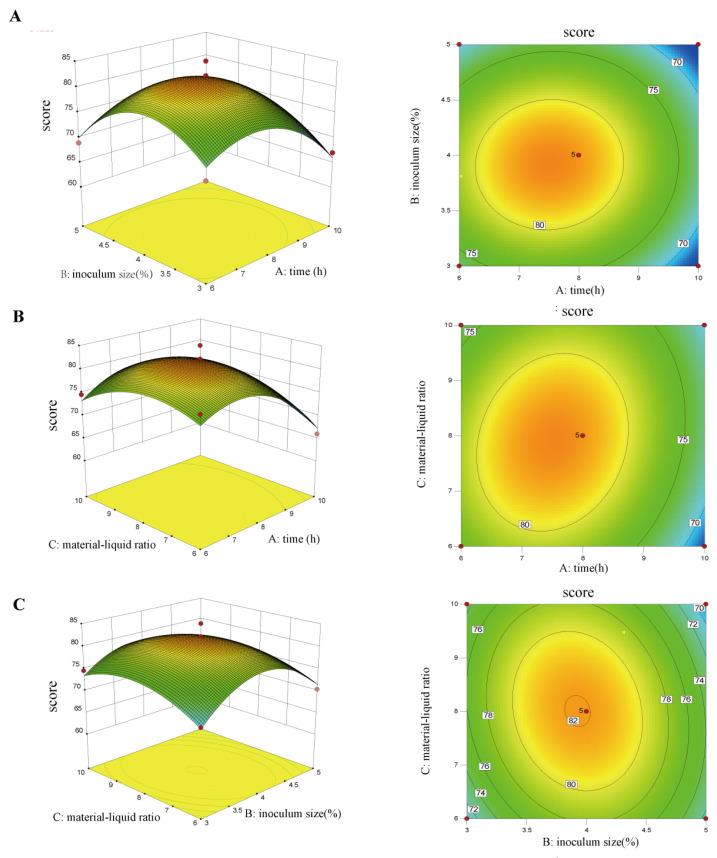
A 3D response surface and contour plots of the interaction between different factors. (**A**) Response surface and contour map of the influence of fermentation time and liquid-solid ratio on the comprehensive score; (**B**) Response surface and contour map of the influence of fermentation time and liquid–solid ratio on the comprehensive score; (**C**) Response surface and contour map of the influence of inoculum size and liquid–solid ratio on the comprehensive score.

Furthermore, the 3D response surface and contour plots of the interaction between different factors were illustrated in Appendix A, Figure A1. From the 3D response surface plots, it could be seen that the interaction was AC > BC > AB, indicating that the fermentation time and liquid ratio had the most significant effect on the beverage fermentation. Taken together, the optimal fermentation process conditions for the fermentation of goji beverage by triple probiotics were obtained as follows: the liquid-to-feed ratio was 7.91; the inoculum amount was 3.92%; the fermentation time was 7.49 h; the fermentation temperature was 37 °C, and the ratio of the triple-probiotic was 1:1:2. Finally, the validation tests under these conditions were carried out, and the actual value of the composite score was 83, which was close to the predicted value of the model, proving that the model was stable and reliable, and the resulting fermented beverage was rich in viable bacteria and high in antioxidant properties.

### 3.2. Analysis of Flavor Substances of Triple-Probiotic-Fermented Goji Beverages

Aroma components, though present in trace amounts, significantly influence the flavor profile and market competitiveness of probiotic-fermented beverages [25]. Importantly, some bioactive compounds in these beverages may directly or indirectly contribute to the therapeutic effects on HUA, while the optimization of fermentation conditions can further enhance both their aromatic characteristics and potential health benefits. Compounds such as alcohols, acids, ketones, and aldehydes were analyzed and identified, and the results are shown in Appendix A, Table A1. As shown in Appendix A, Table A1, 17 kinds of flavor substances, including 8 kinds of alcohols, 1 kind of acid, 4 kinds of ketones, and 4 kinds of aldehydes, were detected in the goji juice. A total of 19 kinds of flavor substances, including 9 kinds of alcohols, 2 kinds of acids, 4 kinds of ketones, and 4 kinds of aldehydes, were detected in the triple-probiotic-fermented goji beverage. This indicated that the types and contents of flavor substances of goji were significantly increased after fermentation with triple probiotics. The relative peak area of alcohols in the beverage was 20.27%, in which the most contained alcohols were n-hexanol with slight wine and fruit aroma; the relative peak area of acids was 5.91%; the most contained acid was acetic acid, which could give the fermented beverage a sweet and sour taste; the relative peak area of ketones was 26.6%; the most abundant ketone was 2,3-butanedione, which had a light milky and sweet aroma; the relative peak area of aldehydes was 5.97%, and the most abundant aldehydes was decanal, which was capable of presenting the fruity aroma of vanilla and sweet orange. In addition, 1-octen-3-ol had a special aroma of mushroom; 2-undecanol had a light sweet aroma; phenylethanol had a pleasant rose aroma; 3-hydroxy-2-butanone had a characteristic milk aroma; n-octanal had a jasmine and grassy aroma, and β-violet ketone had a special hyacinth aroma, which were all crucial to the flavor of the triple-probiotic-fermented juice. Notably, acetic acid and 2,3-butanedione showed significant increases after probiotic fermentation (5.53-fold and 4.46-fold higher, respectively). While acetic acid (SCFA) may indirectly influence uric acid excretion through gut microbiota modulation or renal transporters like URAT1 and 2,3-butanedione (ketone) might have potential metabolic links through its association with energy metabolism disorders often comorbid with hyperuricemia, we acknowledge that direct evidence remains limited [26,27].

### 3.3. Effects of Goji Juice and Probiotic-Fermented Goji Beverages on HUA In Vitro and In Vivo

**Figure 2:**
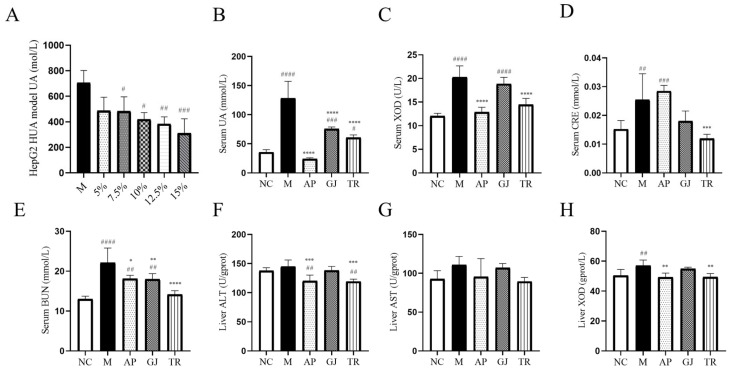
Effects of goji juice and probiotic-fermented goji beverages on HUA cells and mouse models. (**A**) UA level in HepG2 cell model; (**B**) Serum UA level; (**C**) Serum XOD activity; (**D**) Serum CRE level; (**E**) Serum BUN level; (**F**) Liver ALT level; (**G**) Liver AST level; (**H**) Liver XOD activity. * or ^#^ *p* < 0.05, ** or ^##^ *p* < 0.01, *** or ^###^ *p* < 0.001, **** or ^####^ *p* < 0.0001 (^#^ vs. NC and * vs. M group). NC was normal control group. M was HUA model group. AP was the group treated with allopurinol. GJ was goji juice. TR was triple-probiotic-fermented goji beverage (*L. plantarum* TCCC11824, *L. lactis* LTJ28, *L. casei* YR2-2).

HepG2 cells induced by adenosine and xanthine oxidase were treated with 10% triple-probiotic-fermented beverages. The effects on the intracellular UA content are presented in Figure 2A. The elevated intracellular UA content in the M group indicated that the adenosine and xanthine oxidase-induced HUA cell model was successfully established. The content of intracellular UA within each experimental group showed different degrees of reduction compared to the M group. The UA levels in the 5% and 7.5% beverage groups were reduced by 31%, 40.4% in the 10% beverage group, 45.6% in the 12.5% beverage group, and 56% in the 15% beverage group. The cellular UA level was significantly reduced (40.4% and 45.6%, *p* < 0.05) after treatment with 10% and 12.5% fermented beverages, respectively, and significantly (*p* < 0.01) reduced in the 15% fermented beverage group by 56% compared to group M. The UA level was significantly reduced (*p* < 0.05) in the 15% fermented beverage group. The above results indicated that 10–15% fermented beverage of goji was able to inhibit the adenosine and xanthine oxidase-induced elevation of UA content in hepatocytes.

XOD is a key rate-limiting enzyme in purine metabolism, capable of converting hypoxanthine to xanthine in the liver and then transforming xanthine to UA [28,29]. BUN and CRE are two important indicators for the clinical evaluation of renal function impairment [30]. As shown in Figure 2B, goji juice and triple-probiotic-fermented beverages significantly reduced the UA level of HUA mice (*p* < 0.0001), indicating the success of the animal test modeling. As shown in Figure 2C, the XOD level of the mice in group M was significantly higher than that of the mice in group NC (*p* < 0.0001), and as shown in Figure 2C; the BUN and CRE levels of the mice in group M were significantly higher than that of the mice in group NC (*p* < 0.05), suggesting that the high-purine diet in combination with the potassium oxalate modeling method seriously affected the UA metabolism of the mice and caused damage to their metabolic system. Goji juice and a triple-probiotic-fermented beverage were able to reduce xanthine oxidase activity and alleviate impaired renal function. In addition, triple-probiotic-fermented goji beverage could significantly reduce the HUA-induced elevation of BUN and CRE (*p* < 0.0001) (Figure 2D,E). These findings suggested that both goji and fermented beverages had the effect of tonifying the kidneys, and the functional substances of goji that exert UA-lowering and anti-inflammatory effects might have been strengthened after the probiotic fermentation treatment.

AST and ALT are mainly found in the cytoplasm of the liver and are commonly used clinically to evaluate liver function impairment [31]. Liver homogenates from mice were assayed for AST, ALT, and XOD, as shown in Figure 2F–H. The enzyme activities of AST, ALT, and XOD were increased in group M mice compared with those in the NC group. However, while unfermented goji shows only weak effects on these three enzymes, the probiotic-fermented goji beverage can effectively inhibit the activity of these enzymes (especially ALT and XOD).

### 3.4. Probiotic-Fermented Goji Beverage Alleviates HUA-Induced Hepatic, Renal, and Intestinal Damage by Modulating Pathological Changes

Previous studies have indicated that the effects of HUA on hepatic and renal injury are multifaceted, including promotion of urate crystal deposition, endothelial cell damage, inflammatory response, insulin resistance, and direct inhibition of insulin signaling, which, together, may lead to further deterioration of renal function and liver injury [32]. The effects of HUA on intestinal damage are multifaceted and include dysbiosis of the intestinal flora, impaired intestinal barrier function, and inflammatory responses [33]. HUA often causes atrophy of organs such as kidneys and spleens in mice and can trigger metabolic syndrome, such as obesity, and thus, liver hypertrophy [34]. As shown in Table 6, compared with the NC group mice, the body weight and liver index of the M group mice increased (*p* < 0.01). The results of the GJ and TR groups showed a decrease in the liver index and a non-significant change in the renal index after the intervention of goji juice and the triple-probiotic-fermented goji beverage. This indicated that the means of a high-purine diet combined with injection of potassium oxalate could lead to liver and kidney function damage in mice and that goji juice and triple-probiotic-fermented goji beverage had a protective effect on the organs of mice with HUA.

**Figure 3:**
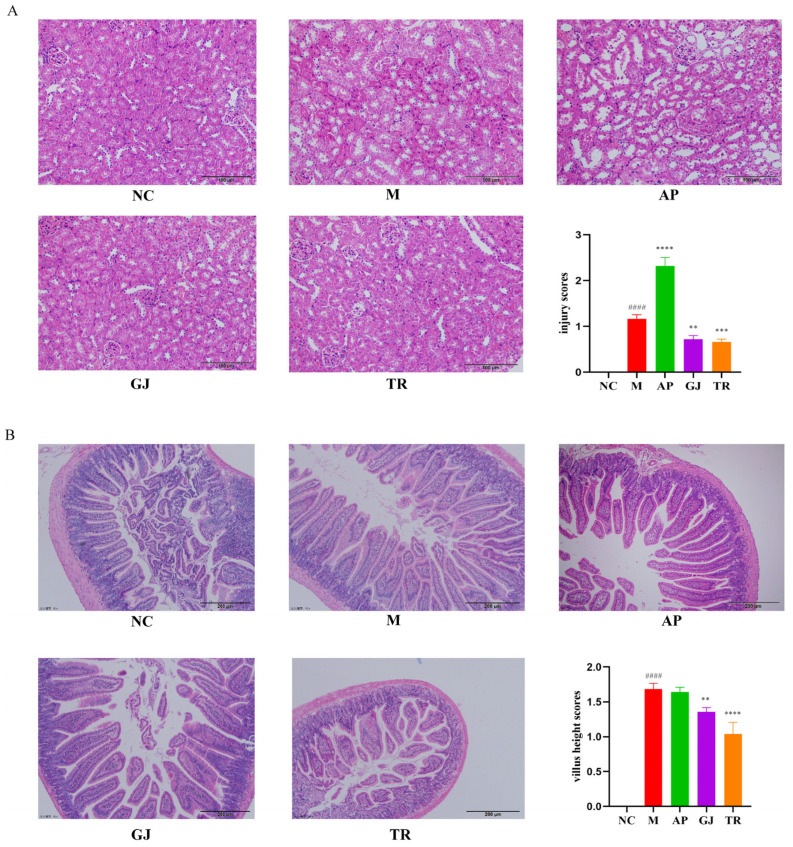
Histopathological analysis of goji juice and probiotic-fermented goji beverages on HUA mice. (**A**) Staining of kidney sections (200×); (**B**) Staining of small intestine sections (100×). NC was normal control group. ** *p* < 0.01, *** *p* < 0.001, **** or ^####^ *p* < 0.0001 (^#^ vs. NC and * vs. M group). M was HUA model group. AP was the group treated with allopurinol. GJ was goji juice. TR was triple-probiotic-fermented goji beverage (*L. plantarum* TCCC11824, *L. lactis* LTJ28, *L. casei* YR2-2).

In order to assess the protective effect of goji juice and triple-probiotic-fermented goji beverage on kidney injury in HUA mice, HE staining was performed on kidney sections. As shown in Figure 3A, the glomerular structure of NC group mice was intact; the renal tubules were clearly visible, and the renal cells were arranged more neatly; in the mice of group M, the nuclei of the renal tissues were aggregated, and there was infiltration of renal interstitial inflammatory cells, as well as dilatation of the distal renal tubules, lumen increase, and a tendency toward vacuolization in the tubular epithelial cells; compared with group M, there was a significant decrease in the number of vacuolated cells, and the renal tissue structure also basically tended to be normal. The renal tissue structure was also basically normalized. This suggested that both goji juice and probiotic-fermented beverages could improve kidney tissue and cell morphology to different degrees and protect the kidneys. In addition, the kidney sections of mice in the AP group showed severe vacuolization of renal tubular epithelial cells. This finding suggests that the current drugs used to alleviate HUA and gout may have an impact on the metabolism of the organism, which is consistent with the literature reports.

Approximately 70% of UA in the body is excreted through the kidneys and 30% through the intestine [30]. In order to detect the effect of HUA on intestinal tissues, HE staining was performed on small intestinal sections from different groups of mice. As shown in Figure 3B, the small intestinal villus tissue of NC group mice was morphologically normal and free of breakage, and the tips of villi of HUA mice induced by a high purine diet combined with potassium oxalate showed different degrees of breakage. Goji juice and a triple-probiotic-fermented goji beverage improved the breakage of the villi of the small intestines. The comparable extent of intestinal mucosal damage observed in the AP and M mouse groups further corroborates the potential hazards associated with the side effects of anti-gout medications, such as allopurinol [32].

### 3.5. Probiotic-Fermented Goji Beverage Attenuates UA Reabsorption Through GLUT9 and URAT1 Regulation

**Figure 4:**
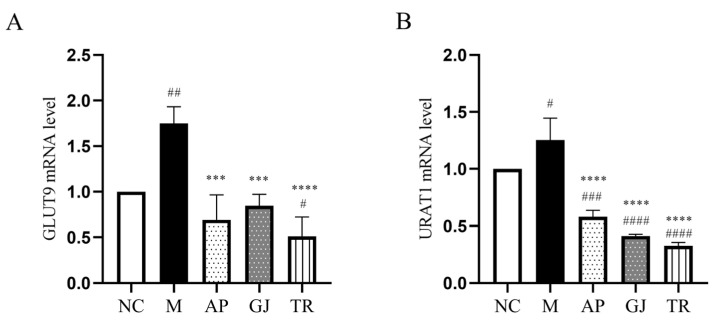
Effects of goji juice and probiotic-fermented goji beverages on transcription of UA transport-related genes in the kidneys of mice in each group. (**A**) GLUT9 mRNA level; (**B**) URAT1 mRNA level. Data are expressed as mean ± SD (n = 10). ^#^ *p* < 0.05, ^##^ *p* < 0.01, ^###^ or *** *p* < 0.001, ^####^ or **** *p* < 0.0001 (^#^ vs. NC and * vs. M group). NC was normal control group. M was HUA model group. AP was the group treated with the allopurinol. GJ was goji juice. TR was triple-probiotic-fermented goji beverage (*L. plantarum* TCCC11824, *L. lactis* LTJ28, *L. casei* YR2-2).

The major excretion of UA in the kidney involves both secretion and reabsorption in the kidney, with almost 90% of UA being reabsorbed back into the bloodstream in the renal tubules, and this reabsorption process is mainly regulated by the urate transport proteins [34]. GLUT9 and URAT1 are the main proteins responsible for the regulation of UA transport, which act on the epithelium of the renal tubule to inhibit tubular reabsorption [35]. Regulating the expression level of urate transporter proteins may affect UA excretion, which, in turn, affects blood UA levels [36]. From Figure 4A, the mRNA level of renal GLUT9 was elevated in mice of group M compared with group NC, which was improved to different degrees after the intervention of goji juice and goji probiotic-fermented beverage. Among them, the triple-probiotic-fermented goji beverage decreased the mRNA level of GLUT9 in the kidneys of mice (*p* < 0.0001). As shown in Figure 4B, the mRNA transcription level of renal URAT1 in the M group of mice was higher than that in the NC group. The goji juice and triple-probiotic-fermented goji beverage could significantly reduce the mRNA level of renal URAT1 in mice (*p* < 0.0001). Therefore, goji juice and goji-fermented beverages can reach the effect of reducing uric acid levels by inhibiting UA reabsorption.

### 3.6. Intestinal Flora Remodeling and Intestinal Barrier Restoration by Probiotic-Fermented Goji Beverage in HUA Mice

HUA can lead to dysbiosis of the intestinal flora, a dysbiosis that has been associated with a variety of renal diseases [37]. Studies suggested that HUA-induced dysbiosis of the gut flora may contribute to the development of kidney injury by promoting the production of enteric-derived uremic toxins and subsequent activation of NLRP3 inflammatory vesicles [38]. This intestinal dysbiosis may also affect intestinal barrier function, increasing intestinal permeability and leading to endotoxin translocation, which, in turn, triggers or exacerbates systemic inflammation [39]. HUA can lead to impaired intestinal barrier function, which may be associated with intestinal dysbiosis. Impairment of intestinal barrier function may lead to easier entry of harmful substances from the gut into the circulation, thus affecting systemic health [40]. Intestinal flora is involved in the catabolism of purines and UA, which may reduce the absorption of purines and UA from the gut, thus alleviating the symptoms and reducing the risk of HUA [41]. As shown in Table 7, Shannon’s index was reduced in all groups of group M compared with group NC, and Shannon’s index was improved by instilling fermented goji beverage; the results of flora diversity indicated that HUA impaired the diversity and richness of intestinal flora, and the goji-fermented beverage could promote the recovery of intestinal flora. However, this change in indices was not very significant, perhaps due to the short modeling time. In addition, it has been reported that diet is also a key factor in shaping the intestinal flora, i.e., the groups of mice fed the same food and water showed fewer differences in their intestinal microecology [42].

**Figure 5:**
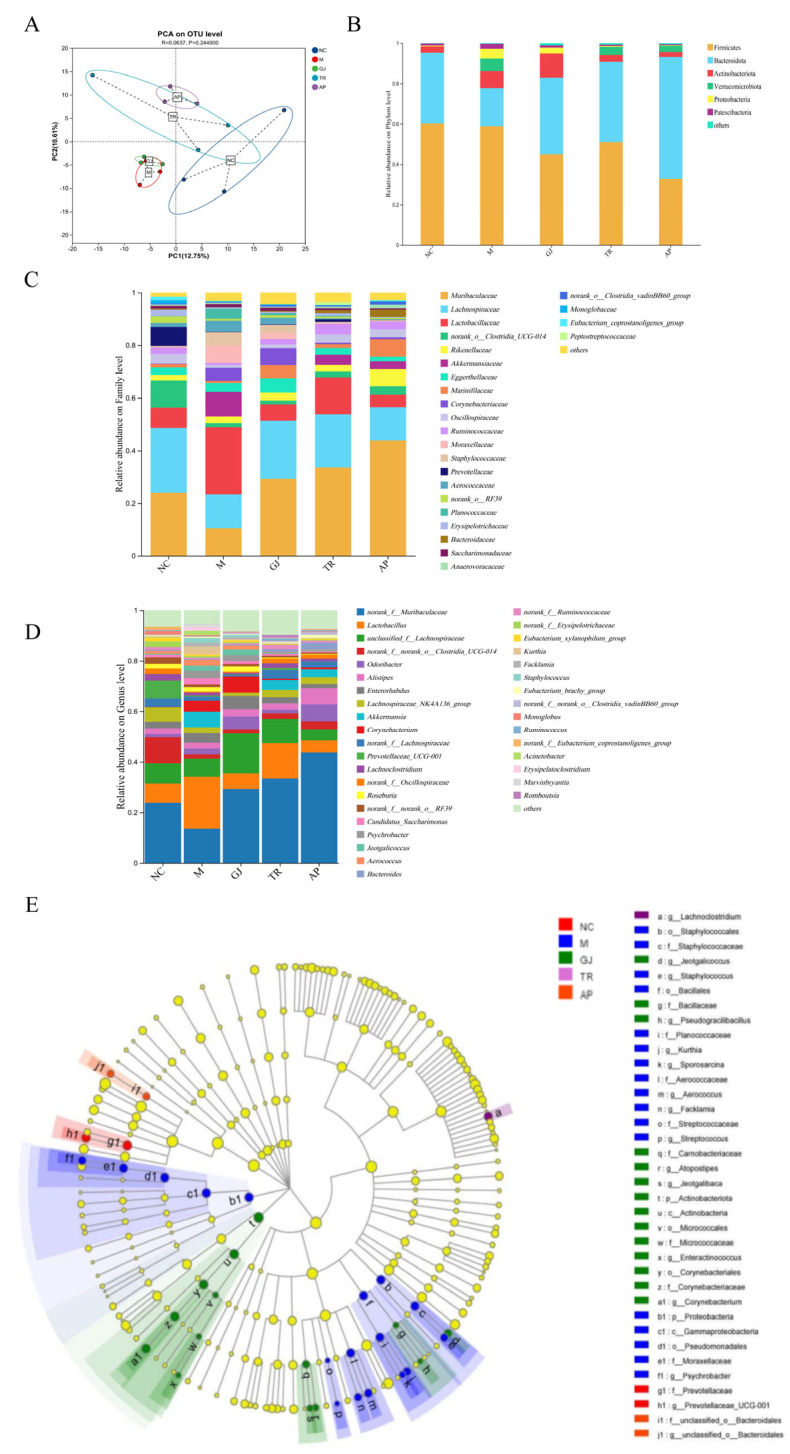
Effects of goji juice and probiotic-fermented goji beverages on intestinal flora of HUA mice. (**A**) Analysis of principal coordinates based on the OTU level; (**B**) The species composition of intestinal flora at the Phylum level; (**C**) The species composition of intestinal flora at the Family level; (**D**) The species composition of intestinal flora at the Genus level; (**E**) Hierarchical Tree Map of Lefse Multi-level specie. NC was normal control group. M was HUA model group. AP was the group treated with the allopurinol. GJ was goji juice, TR was triple-probiotic-fermented goji beverage (*L. plantarum* TCCC11824, *L. lactis* LTJ28, *L. casei* YR2-2).

From the PCoA analysis in Figure 5A, the difference in colony structure between the NC group and the M group was large, and the other groups of mice could improve the colony structure of mice to different degrees compared with the M group. Principal coordinates analysis revealed distinct clustering patterns: the GJ group’s microbiota composition closely resembled that of the M group, whereas the fermented goji beverage group exhibited significant separation from the M group and closer proximity to the NC group. In addition, it was further shown that in this animal test, the intestinal flora of HUA mice was less affected by the infusion of goji alone, and the intestinal flora structure of HUA mice could be improved after being prepared as a beverage by probiotic fermentation. Based on the OTU data, gut microorganisms were classified at the phylum level, as shown in Figure 5B. The composition of the flora at the phylum level in each group of mice was mainly composed of *Bacteroidetes*, *Firmicutes*, *Actinobacteriota*, *Verrucomicrobia*, *Proteobacteria*, and *Patescibacteria*, which are six phyla, in addition to unclassified bacteria. Compared with the colony abundance of Mycobacterium anisopliae in the NC group (with a percentage of 35.14%), it was significantly lower in the M group (with a percentage of 25.93%); whereas the GJ, TR, and AP groups all significantly increased the abundance of *Mycobacterium* anisopliae compared to the model group, with the GJ group with a percentage of 37.95%, the TR group with a percentage of 39.77%, and the AP group with a percentage of 60.38%. In addition, the abundance of thick-walled bacilli phylum was significantly lower in the M group compared to the NC group (with a percentage of 60.21%). Similarly, the GJ and TR groups could significantly increase the abundance of the thick-walled bacilli phylum compared to the model group, with 45.03% share in the GJ group and 51.04% share in the TR group. The anabaena phylum and thick-walled phylum flora are responsible for promoting the body’s absorption of energy from dietary sources, and together, they maintain the energy metabolism of the body and the stable operation of intestinal physiological functions. In animal experiments, the yeast paste combined with potassium oxonate induced a decrease in the abundance of Bacteroides uniformis and other Firmicutes bacteria in the M group of mice. And after 28 d of intervention, *Bacteroides* spp. with abnormal abundance and the Firmicutes phylum were increased, suggesting that this may be a result of the goji-fermented beverage regulating and improving the structure of the intestinal dominant bacterial flora and promoting the restoration of a normal intestinal micro-ecological environment. This suggests that the goji-fermented beverage has a significant effect on the dysregulation of intestinal flora in HUA mice. These results indicate that the goji-fermented beverage might have a certain effect on improving and regulating the dysbiosis of intestinal flora in HUA mice.

At the family level, as shown in Figure 5C, the main constituent bacteria of the intestinal flora of the five groups of mice at the family classification level were *Muribaculaceae*, *Lachnospiraceae*, *Lactobacillaceae*, *norank_o_Clostridia_UCG-014*, *Rikenellaceae*, *Marinifilaceae*, *Oscillospiraceae*, *Eggerthellaceae*, *Ruminococcaceae*, and *Corynebacteriacea*, which are 10 families of bacteria. Among them, *Muribaculaceae* and *Lachnospiraceae* are the two most dominant. Compared with the abundance of Mycobacteriaceae in the NC group (with a percentage of 23.94%), it was significantly lower in the M group (with a percentage of 18.84%), whereas the GJ, TR, and AP groups showed different degrees of increase compared with the model group, with a percentage of 29.27% in the GJ group, 33.52% in the TR group, and 43.85% in the AP group. The abundance of triple-probiotic-fermented beverages was also significantly lower in the M group compared to the NC group (with a percentage of 24.69%). Previous studies have shown that *Anabacteriaceae*, the dominant family under the phylum *Anabacteria* in the mouse intestine, is highly functional in degrading complex carbohydrates, and, thus, *Anabacteriaceae* abundance tends to decrease in the intestinal flora of obese mice fed with high-calorie diets [43]. As for *Trichoderma*, its abundance has been reported to be negatively correlated with blood pressure and cholesterol levels, and in the present study, the reduced abundance of *Anabaena* and *Trichoderma* in the HUA model group of mice revealed the relevance of HUA to obesity as well as cardiovascular disease [44].

Species composition analysis at the genus level is shown in Figure 5D, in which the top three homogeneous genera with more significant changes were *norank_f_Muribaculaceae*, *Lactobacillus*, and *unclassified_f_Lachnospiraceae*, respectively. Compared with the NC group, the relative proportion of the M group, *norank_f_ Muribaculaceae* (with a percentage of 18.84%), decreased, while the GJ group and TR group both had different degrees of elevation. Muribaculaceae (with a percentage of 18.84%) showed a decrease in relative percentage, while the GJ and TR groups showed different degrees of increase. Compared with group M, the abundance of *Lactobacillus* in group TR was 2.14 times higher than that in group M. The probiotic-fermented goji beverage significantly increased the abundance of the beneficial gut bacterium *Lactobacillus spp.* in mice. This indicates that fermented beverages with live bacteria may have better physiological functions. Overall, the results suggest that probiotic-fermented goji beverages can treat HUA by regulating the intestinal flora.

A total of 15 different species were found at the genus level, as can be seen in Figure 5E. It can be seen that group M was significantly enriched in *p_Proteobacteria*, and group GJ was significantly enriched in *p_Actinobacteriota*. It has been shown that the *Ascomycota* disrupts the anaerobic equilibrium state of the intestines, and that upregulation of *Actinobacteria* abundance is also a significant contributor to the differential effect of the intestinal flora [45]. Upregulation is also an important feature of intestinal dysbiosis, and in this study, HUA disrupted the anaerobic environment in the mouse colon, resulting in dysbiosis and metabolic disorders. This dysbiosis was not improved by the intake of goji alone. The abundance of *Clostridium* is generally negatively correlated with the degree of enteritis, such as *Clostridium typhimurium ATCC25755*, which exerts anti-inflammatory and intestinal homeostatic effects [46]. Although whether *Clostridium* plays a role within the intestinal environment of HUA patients has not been reported, it is clear from the combination of the results of the present study that probiotics played a positive role in the intestinal flora of the HUA model and that the probiotic-fermented goji beverage could regulate the intestinal flora and exert its homeostatic function in HUA mice.

### 3.7. Probiotic-Fermented Goji Beverage Modulates Gut Microbiota-Derived SCFAs in HUA

**Figure 6:**
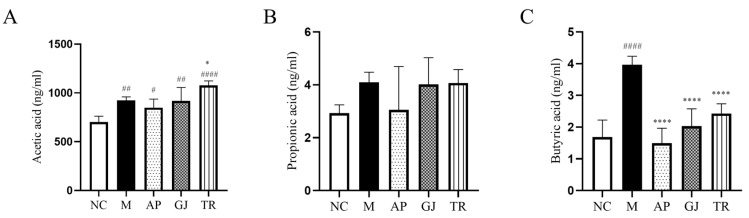
Effects of goji juice and probiotic-fermented goji beverages on colonic SCFAs content in mice. (**A**) Acetic acid level; (**B**) Propionic acid level; (**C**) Butyric acid level. Data are expressed as mean ± SD (n = 10). * or ^#^ *p* < 0.05, ^##^ *p* < 0.01, **** or ^####^ *p* < 0.0001 (^#^ vs. NC and * vs. M group). NC was normal control group. M was HUA model group. AP was the group treated with the allopurinol. GJ was goji juice. TR was triple-probiotic-fermented goji beverage (*L. plantarum* TCCC11824, *L. lactis* LTJ28, *L. casei* YR2-2).

Metabolites (SCFAs) of the intestinal flora are associated with UA excretion and can influence UA levels in the host, and therapies targeting the intestinal flora may be a new target for controlling the pathogenesis of HUA [47]. SCFAs are major metabolites of intestinal flora, including acetic acid, propionic acid, and butyric acid. Studies have shown that acetic acid is an important ligand in the inflammatory response to gout, and a deficiency of acetic acid leads to low immunity in mice [48]; propionic acid inhibits intracellular lipid degradation and increases the lipid buffering capacity of adipose tissues; butyric acid is essential for the maintenance of the integrity of the intestinal barrier [33]. However, recent findings suggest that SCFAs, such as acetic acid, while beneficial to host health, are equally harmful at high levels [49]. The effects of goji juice and goji probiotic-fermented beverage on HUA mice were investigated by examining the levels of the three SCFAs in the colon contents, and the results are shown in Figure 6A–C. Compared with the NC group of mice, group M mice showed a significant increase in the levels of acetic acid and butyric acid in the colon (*p* < 0.001; *p* < 0.0001) and propionic acid in the colon, with no statistically significant difference. The butyric acid content of the HUA mice was significantly reduced (*p* < 0.0001) by the intervention of goji juice and triple-probiotic-fermented beverage and converged to the level of the NC group. As for propionic acid contents, the effects of goji juice and fermented beverages were not significant. Goji juice and goji probiotic-fermented beverages significantly increased the acetic acid content of cecum contents in mice (*p* < 0.001; *p* < 0.0001). Importantly, the restoration of butyrate levels to near NC group following goji probiotic-fermented beverage intervention (*p* < 0.0001) suggests a protective effect on gut health, consistent with its known function in strengthening the intestinal barrier. While acetic acid levels rose significantly (*p* < 0.0001), they remained within ranges reported in prior studies. The increased abundance of SCFAs in the intestinal tract of mice treated with the goji probiotic-fermented beverage suggests a potential improvement in the gut microenvironment, which is associated with intestinal barrier function and overall gut health.

## 4. Conclusions

This research established for the first time a fermentation process for a goji-fermented beverage by triple probiotics (*L. plantarum* TCCC11824, *L. lactis* LTJ28, *L. casei* YR2-2) and investigated its effects on HUA at cellular and animal levels. The effects of goji juice, as well as the triple-probiotic-fermented goji beverage, on HUA mice were also compared. The optimal process conditions of the triple-probiotic-fermented beverage were as follows: liquid-to-feed ratio of 7.913, inoculum amount of 3.92%, fermentation time of 7.49 h, fermentation temperature of 37 °C, and the ratio of *L. plantarum* CGMCC8198: *L. lactis* LTJ28: *L. casei* YR2-2 strains of 1:1:2. The content and types of flavoring substances of the triple-probiotic-fermented beverage increased significantly compared with those of the unfermented goji juice.

Triple-probiotic-fermented beverages could reduce UA production in the HUA cell model of HepG2. Among them, the 15% beverage treatment led to the most significant reduction, with the UA value being reduced by 56% compared to the HUA model. Goji juice and a triple-probiotic-fermented beverage could effectively reduce the liver index and increase the kidney index in HUA mice. Compared with the HUA model, goji juice and triple-probiotic-fermented beverage could reduce the levels of serum UA, BUN, CRE, XOD, and liver XOD, AST, and ALT levels and regulate the metabolic disorders of renal and hepatic functions induced by HUA. However, the effect of triple-probiotic-fermented beverage on improving uric acid metabolism was better than other groups; goji juice and triple-probiotic-fermented beverage could improve the abundance of SCFAs such as acetic, propionic, and butyric acids in the colons of the mice with HUA, and maintain the health of intestinal barrier; goji juice and triple-probiotic-fermented beverage were able to downregulate GLUT9 and URAT1 genes in HUA mice, and the downregulation trend of triple-probiotic-fermented beverage was greater than that of other experimental groups, thus promoting uric acid excretion and improving nephritis and intestinal mucosal injury. Goji juice and a triple-probiotic-fermented beverage improved intestinal species diversity and augmented the relative abundance of beneficial flora, including *Mycobacterium anisopliae*, *Mycobacterium thickum*, *Mycobacteriophage*, and *Trichosporonaceae*.

In conclusion, the triple-probiotic-fermented goji juice demonstrated modulatory effects on the intestinal microecological environment in HUA mice, suggesting its potential as a novel strategy for HUA management at the microbial level. These results indicate a promising approach for HUA intervention; however, further clinical studies are necessary to validate these findings and evaluate their therapeutic efficacy in human applications. This study highlights the potential of probiotic-fermented functional foods as a natural therapeutic approach for managing HUA and associated metabolic disorders.

## Figures and Tables

**Table 1:** Evaluation standard of goji fermentation process.

Evaluation Indicators and Scores	Evaluation Criteria	Scores
Viable bacteria count (50)	10^10^ CFU/mL < Viable bacteria count ≤ 5 × 10^10^ CFU/mL	40–50
	5 × 10^9^ CFU/mL < Viable bacteria count ≤ 10^10^ CFU/mL	30–40
	10^9^ CFU/mL < Viable bacteria count ≤ 5 × 10^9^ CFU/mL	20–30
	5 × 10^8^ CFU/mL < Viable bacteria count ≤ 10^9^ CFU/mL	10–20
	10^8^ CFU/mL < Viable bacteria count ≤ 5 × 10^8^ CFU/mL	0–10
Oxidation resistance (50)	45% < clearance rate ≤ 50%	40–50
	40% < clearance rate ≤ 45%	30–40
	35% < clearance rate ≤ 40%	20–30
	30% < clearance rate ≤ 35%	10–20
	25% < clearance rate ≤ 30%	0–10

**Table 2:** Three-factor and three-level table of fermentation process.

Level	Factor		
	A Fermentation Time	B Vaccination Load	C Material–Liquid Ratio
−1	6 h	3%	6
0	8 h	4%	8
1	10 h	5%	10

**Table 3:** Primer sequence table for real-time PCR.

Gene	Sequence (5′→3′)
GAPDH	ATGGTGAAGGTCGGTGTGAACGG
	TGGAACATGTAGACCATGTAGTTGAGG
URAT1	TCCTGAACTCCTGGACCGAGTG
	AGTTCTCCAGCATGTTCTGA
GLUT9	GTGAAAAGAACTCCGCAGAAACCA
	AGGAAGGAGGACCCGAAGGCTC

**Table 4:** Response surface experiment design and results scheme table.

Serial Number	Factor			
	A Fermentation Time (h)	B Inoculation Amount (%)	C Material–Liquid Ratio	Score
1	6	3	8	70.8
2	6	5	8	69
3	8	3	6	71
4	10	5	8	67.2
5	6	4	10	74.6
6	8	4	8	80
7	8	5	6	70.4
8	6	4	6	78.6
9	8	3	10	74.6
10	10	3	8	67
11	10	4	10	68
12	8	4	8	82.2
13	8	4	8	82
14	8	4	8	85
15	10	4	8	66
16	8	4	8	81
17	8	5	10	68.2

**Table 5:** The results of the significance test of the regression coefficients of the response surface model.

Source of Variance	SS	DoF	Variance	F	*p*	Significance
Model	602.62	9	66.96	11.10	0.0022	** (*p* < 0.01)
A	76.88	1	76.88	12.74	0.0091	** (*p* < 0.01)
B	9.24	1	9.24	1.53	0.2557	
C	0.045	1	0.045	7.457 × 10^−3^	0.9336	
AB	1	1	1	0.17	0.6961	
AC	9	1	9	1.49	0.2615	
BC	8.41	1	8.41	1.39	0.2763	
A^2^	172.19	1	172.19	28.53	0.0011	
B^2^	214.95	1	214.95	35.62	0.0006	
C^2^	62.25	1	62.25	10.32	0.0148	
Residuals	42.24	7	6.03			
Ack of fit	28.21	3	9.40	2.68	0.1823	
Pure error	14.03	4	3.51			
Total deviation	644.86	16				

**Table 6:** Effects of goji juice and probiotic-fermented goji beverages on body weight and organ index of animal ($\bar{x}$ ± n, *n* = 10; ** *p* < 0.01).

Group	Weight (g)	Liver Index (%)	Kidney Index (%)
NC	35.75 ± 2.92	3.58 ± 0.57	1.34 ± 0.18
M	36.05 ± 2.58	4.28 ± 0.39 **	1.24 ± 0.15
AP	36.28 ± 2.08	4.80 ± 0.64	1.37 ± 0.21
GJ	36.7 ± 1.78	4.24 ± 0.21	1.10 ± 0.07
TR	36.6 ± 2.42	4.18 ± 0.40	1.24 ± 0.11

**Table 7:** Species diversity and richness of mouse colon contents.

Graph	Simpson	Shannon	Chao	Ace
NC	0.04 ± 0.01	4.18 ± 0.13	382.88 ± 19.54	384.21 ± 15.29
M	0.06 ± 0.03	3.83 ± 0.36	383.42 ± 13.08	385.34 ± 9.02
AP	0.03 ± 0.01	4.26 ± 0.11	414.63 ± 7.14	406.87 ± 8.60
GJ	0.06 ± 0.01	3.81 ± 0.13	385.92 ± 4.95	380.82 ± 1.39
TR	0.05 ± 0.01	4.07 ± 0.10	411.3 ± 45.10	404.41 ± 46.39

## Data Availability

The original contributions presented in this study are included in this article; further inquiries can be directed to the corresponding authors.

## Appendix A

**Table A1:** Volatile flavor components and relative content before and after fermentation.

Compound Type	Chemical Compound	CAS	Relative Amount in Goji Juice (%)	Relative Amount in Triple-Probiotic-Fermented Goji Beverage (%)
alcohol compound	3-Methyl-1-butanol	123-51-3	0.44%	0.33%
	n-Hexyl alcohol	111-27-3	18.01%	13.5%
	undecan-2-ol	1653-30-1	1.45%	1.15%
	1-Octanol	111-87-5	-	0.81%
	Phenethyl alcohol	60-12-8	0.51%	0.5%
	trans-2-hexen-1-ol	928-95-0	12.42%	1.03%
	2-Ethyl-1-hexanol	104-76-7	1%	0.75%
	1-Heptanol	111-70-6	0.19%	0.34%
	1-Octen-3-ol	3391-86-4	1.63%	1.86%
acid compounds	Acetic acid	64-19-7	-	5.53%
	Nonanoic acid	112-05-0	0.8%	0.38%
ketone compound	2,3-Butanedione	431-03-8	2.73%	12.17%
	Acetyl methyl carbinol	513-86-0	18.29%	12.4%
	6-Methyl-5-hepten-2-one	110-93-0	1.32%	1.2%
	beta-ionone	79-77-6	1.28%	0.83%
aldehyde compound	Caproaldehyde	66-25-1	1.07%	0.54%
	Benzaldehyde	100-52-7	0.4%	0.28%
	Octyl aldehyde	124-13-0	0.51%	1.1%
	Decanal	112-31-2	1.77%	4.05%
